# Acupuncture modulates the functional connectivity among the subcortical nucleus and fronto‐parietal network in adolescents with internet addiction

**DOI:** 10.1002/brb3.3241

**Published:** 2023-09-18

**Authors:** Yang Wang, Yun Qin, Hui Li, Dezhong Yao, Bo Sun, Jinnan Gong, Yu Dai, Chao Wen, Lingrui Zhang, Chenchen Zhang, Cheng Luo, Tianmin Zhu

**Affiliations:** ^1^ School of Sports Medicine and Health Chengdu Sport University Chengdu China; ^2^ Postdoctoral Workstation, Affiliated Sport Hospital of Chengdu Sport University Chengdu China; ^3^ School of Rehabilitation and Health Preservation Chengdu University of TCM Chengdu China; ^4^ College of Traditional Chinese Medicine Chongqing Medical University Shapingba China; ^5^ Key Laboratory for NeuroInformation of Ministry of Education University of Electronic Science and Technology of China Chengdu China; ^6^ School of Medicine Chengdu University Chengdu China; ^7^ School of Computer Science Chengdu University of Information Technology Chengdu China; ^8^ Department of Chinese Medicine Chengdu Eighth People's Hospital Chengdu China; ^9^ Department of Rehabilitation Zigong Fifth People's Hospital Zigong China; ^10^ Department of Medicine Leshan Vocational and Technical College Leshan China; ^11^ Department of Rehabilitation TCM Hospital of Longquanyi District Chengdu China; ^12^ Research Unit of NeuroInformation Chinese Academy of Medical Sciences Beijing China; ^13^ Library, Chengdu University of TCM Chengdu China

**Keywords:** acupuncture, internet addiction, large‐scale network, multiple linear regression

## Abstract

**Background:**

Internet addiction (IA), recognized as a behavioral addiction, is emerging as a global public health problem. Acupuncture has been demonstrated to be effective in alleviating IA; however, the mechanism is not yet clear. To fill this knowledge gap, our study aimed to investigate the modulatory effects of acupuncture on the functional interactions among the addiction‐related networks in adolescents with IA.

**Methods:**

Thirty individuals with IA and thirty age‐ and sex‐matched healthy control subjects (HCs) were recruited. Subjects with IA were given a 40‐day acupuncture treatment, and resting‐state functional magnetic resonance imaging (fMRI) data were collected before and after acupuncture sessions. HCs received no treatment and underwent one fMRI scan after enrollment. The intergroup differences in functional connectivity (FC) among the subcortical nucleus (SN) and fronto‐parietal network (FPN) were compared between HCs and subjects with IA at baseline. Then, the intragroup FC differences between the pre‐ and post‐treatment were analyzed in the IA group. A multiple linear regression model was further employed to fit the FC changes to symptom relief in the IA group.

**Results:**

In comparison to HCs, subjects with IA exhibited significantly heightened FC within and between the SN and FPN at baseline. After 40 days of acupuncture treatment, the FC within the FPN and between the SN and FPN were significantly decreased in individuals with IA. Symptom improvement in subjects with IA was well fitted by the decrease in FC between the left midbrain and ventral prefrontal cortex and between the left thalamus and ventral anterior prefrontal cortex.

**Conclusion:**

These findings confirmed the modulatory effects of acupuncture on the aberrant functional interactions among the SN and FPN, which may partly reflect the neurophysiological mechanism of acupuncture for IA.

## Graphical abstract

Functional connectivity network analysis showed that functional interactions among the subcortical nucleus and fronto‐parietal network were significantly heightened in internet addiction, which can be modulated by acupuncture treatment. Furthermore, the decrease of the functional connectivity among these addiction‐related networks by acupuncture can well explain the symptom improvement.

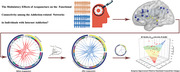

## 1 BACKGROUND

The internet has become a fully integrated part of people's daily lives because of the countless benefits it brings. However, negative consequences have also emerged with the popularization of the internet. Many adolescents indulge in the virtual network, resulting in the impairment of psychological and social functioning, which is known as internet addiction (IA) (Young et al., 1999). With the development of mobile information technology, IA has spread around the world in the last 20 years (Darvesh et al., 2020). Currently, the incidence of IA is approximately 7.02% around the world and is still steeply increasing (Pan et al., 2020). Due to its wide prevalence and high and rapidly growing incidence, IA has been recognized as a serious public health issue (Y. Wang et al., 2021, 2019).

Acupuncture, a traditional Chinese medicine therapy, has been extensively used for various substance addictions (Lee et al., 2021; Nguyen et al., 2021; Shiao et al., 2021). In previous studies, we demonstrated the effectiveness of acupuncture in treating IA. Addictive symptoms were significantly improved after acupuncture treatment in people with internet addictions (Li et al., 2017; Y. Yang et al., 2017). Nevertheless, the mechanism through which acupuncture alleviates IA is not yet clear, and further research on the neuroregulatory effects of acupuncture on IA is necessary.

Aberrant interactions among addiction‐related brain networks are proposed to be the main characteristics of addiction, resonating with the synaptic alterations and morphological adaptation in animal models (Z.‐L. Wang et al., 2023). The extensive application of functional connectivity (FC), allowing for the in vivo investigation of the interactions among large‐scale brain networks, has vastly improved our system‐level understanding of the neuropathogenesis of addiction and the mechanisms of related treatments (Chen et al., 2014). The subcortical nucleus (SN, responsible for primitive instincts) and the fronto‐parietal network (FPN, responsible for cognitive functions) are the most addiction‐associated networks in Yeo's system (Bolmont et al., 2014; Yeo et al., 2011; Yokoyama et al., 2017). Both of the networks are involved in the dual systems model, a generally accepted theory recognizing the dysregulation of cognitive control and reward processing as the essential mechanism of addiction (Shulman et al., 2016). Many FC studies have demonstrated abnormal interactions among the SN and FPN in substance addiction and have revealed the modulatory effects of related therapies on these abnormalities, emphasizing the critical role of FC alterations among the SN and FPN in the pathogenesis of substance addictions (Heilig et al., 2019; Konova et al., 2013; Y. Zhang, et al., 2018). In recent research, altered FC among the SN and FPN was also observed in IA (Zheng et al., 2022; W.‐R. Zhou et al., 2022) and could be modulated by psychotherapy (J.‐T. Zhang et al., 2018), implying the similar role of the interactions among the SN and FPN in IA and substance addiction.

Given that abnormal interactions among the SN and FPN play a vital role in IA and that acupuncture is an effective treatment for internet addicts, we believe that the effects of acupuncture on IA might be associated with FC changes among the SN and FPN. Thus, we designed the current study to explore the neural modulatory effect of acupuncture on FC among the SN and FPN. For this purpose, we investigated the intergroup FC differences between 30 IA adolescents and 30 age‐ and sex‐matched healthy control subjects (HCs) and further explored the intragroup FC changes before and after acupuncture in subjects with IA. Then, multiple linear regression was employed to fit the FC changes to the symptom improvement in individuals with IA. We hypothesized that (1) subjects with IA would exhibit aberrant interactions among the SN and FPN in comparison to HCs, (2) acupuncture could regulate the FC abnormalities among the SN and FPN in individuals with IA, and (3) the FC changes caused by acupuncture would well fit the symptom relief in the IA group.

## 2 MATERIALS AND METHODS

### 2.1 Participants

A total of 60 adolescents aged 18–30 years were recruited (30 HCs and 30 individuals with IA). Each subject with IA met the clinical diagnostic criteria for IA (Beard & Wolf, 2001), while the HCs did not meet the criteria. Symptom severity was evaluated by the Internet Addiction Test (IAT); only individuals with a score of more than 50 were included in the IA group (Y. Wang et al., 2020). Subjects with substantial brain abnormalities or a history of substance dependence were excluded, as well as lactating or pregnant females. All participants signed informed consent forms before enrollment.

### 2.2 Study design

**FIGURE 1:**
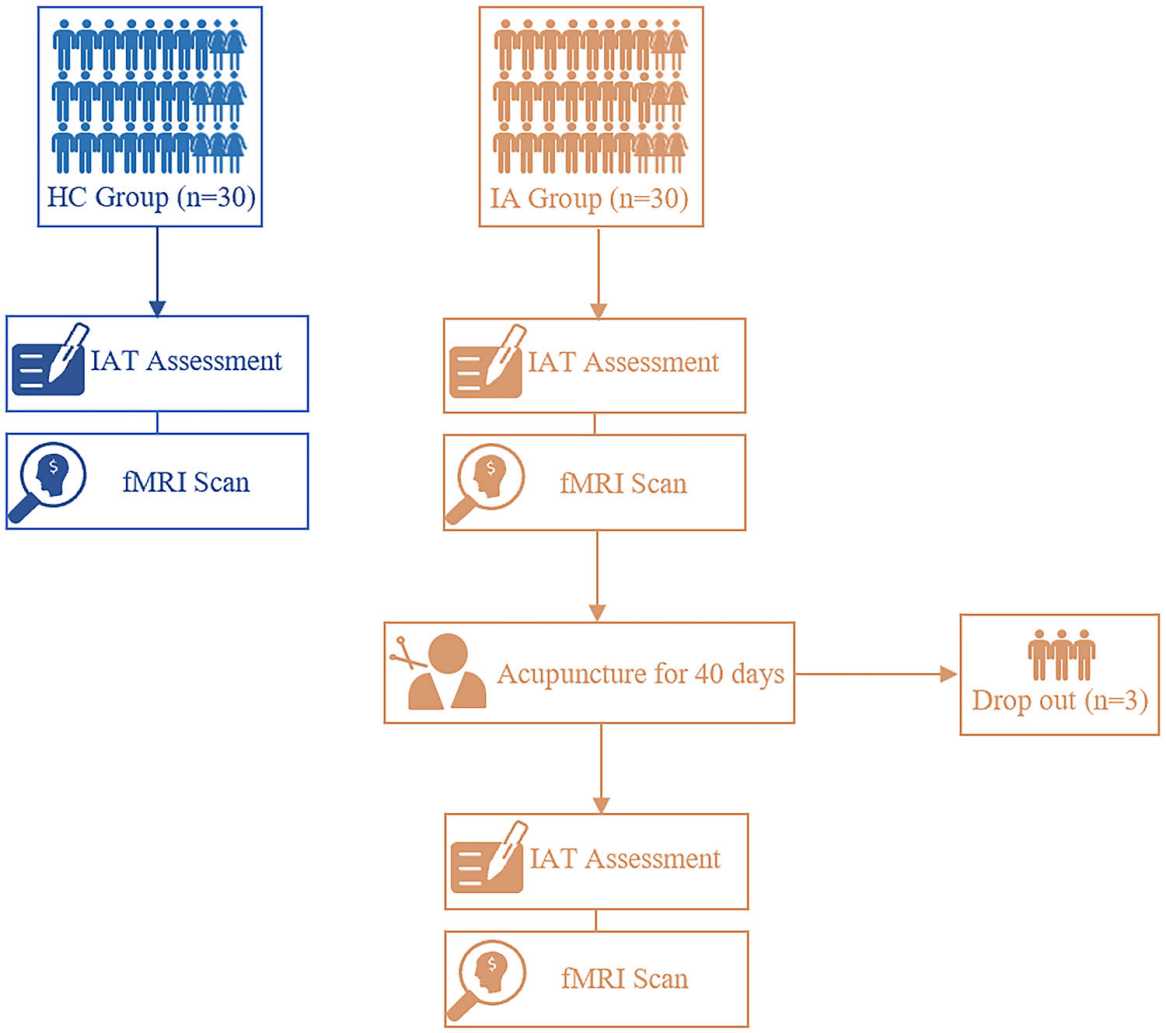
Study flow chart. HC, healthy control; IA, internet addiction; IAT, Internet Addiction Test; fMRI, functional magnetic resonance imaging.

After enrollment, all participants were first assessed using the IAT and then underwent a functional magnetic resonance imaging (fMRI) scan. Subsequently, a 40‐day course of acupuncture treatment was performed for individuals with IA. Three subjects with IA did not complete the acupuncture treatment due to schedule constraints. After acupuncture, the IA group received another clinical assessment and fMRI scan. More detailed procedures are illustrated in Figure 1.

### 2.3 Intervention

The acupuncture treatment for subjects with IA was administered by two licensed acupuncturists. The targeted acupoints were the Sishenchong (EX‐HN1), Baihui (DU‐20), bilateral Sanyinjiao (SP‐6), Neiguan (PC‐6), Taichong (LR‐3), Shenmen (HT‐7), Xuanzhong (GB‐39), and Hegu (LI‐4) (Figure S1). After insertion into the selected acupoints (the depths of needle insertion are shown in Table S1), the stainless‐steel needles (0.30 × 40 mm, Hwato) were lifted and thrust until the individuals felt deqi (a complex and mixed feeling including numbness, distention, soreness, or dull pain at the needling acupoints). Subsequently, the needles were left in the acupoints for 30 min.

The individuals with IA received 20 sessions of acupuncture treatment (30 min each) once every 2 days. The participants were required not to receive any other medical interventions during the research. For HCs, no treatment was performed.

### 2.4 MRI data collection

All fMRI data were collected at the University of Electronic Science and Technology of China using a 3 T MR scanner (GE). During data collection, the subjects were asked to stay awake and clear their minds. Earplugs were employed to minimize noise, and foam pads were used to restrict head movements during scanning.

Functional images were obtained with an echo‐planar imaging sequence (repetition time = 2000 ms, echo time = 30 ms, field of view = 240 mm × 240 mm, voxel size = 3.75 mm × 3.75 mm × 4.4 mm, flip angle = 90°, and image matrix = 64 × 64). Using the sequence, 255 volumes were acquired, with 35 slices contained in each volume.

### 2.5 MRI data processing

The Neuroscience Information Toolbox (Dong et al., 2018) in the MATLAB platform (MathWorks, Inc.) was applied for fMRI preprocessing. First, the volumes obtained in the first 10 seconds were discarded due to the possible inhomogeneity of the major magnetic field. Then, slice timing and spatial realignment were conducted. In spatial realignment, participants with more than 2° rotation or 2‐mm displacement in any direction were eliminated. Afterward, fMRI data were normalized to a standard Montreal Neurological Institution (MNI) template. Head motion parameters and signals from cerebral spinal fluid and white matter were then regressed out. After smoothing (Gaussian kernel of 6 mm), all fMRI data were filtered (bandpass, 0.01–0.08 Hz).

### 2.6 FC network analysis

The FC network was computed by the Graph Theoretical Network Analysis Toolbox (J. Wang et al., 2015). First, the mean BOLD time series of 28 regions of interest (ROIs) in the SN and FPN (seven in the SN and 21 in the FPN) were extracted (Dosenbach et al., 2010; Yeo et al., 2011). Subsequently, the pairwise FC values among all these ROIs (28 nodes with 378 edges) were calculated by correlation analyses. Finally, the resulting correlation coefficients were transformed to Z scores for further parametric analysis.

### 2.7 Statistical analysis

#### 2.7.1 Analysis of clinical outcomes

SPSS 18.0 (SPSS Inc.) was applied for demographic and clinical outcome analysis. For continuous variables, an independent *t* test was conducted to evaluate the intergroup differences between individuals with IA and HCs, while a paired *t* test was applied to analyze the intragroup differences before and after acupuncture in the IA group. The chi‐square test was employed for the analysis of categorical variables. The significance level was set as *p* < .05.

#### 2.7.2 Analysis of FC networks

The intergroup differences in the FC networks between HCs and subjects with IA were compared using independent *t* tests at baseline. The FC network changes caused by acupuncture in the IA group were then analyzed using paired *t* tests. For the fMRI analyses, network‐based statistics (NBS)‐corrected p values of < .05 were defined as significant. In addition, the intergroup and intragroup differences in the average FC strengths (reflecting functional integration) within and between the SN and FPN were also compared.

#### 2.7.3 Multiple linear regression

To investigate the association of FC changes with IA improvement, multiple linear regression was conducted. In the multiple linear regression model, the significant FC changes caused by acupuncture were set as explanatory variables, while IAT score improvement was considered the target variable. The forward stepwise method (alpha level of 0.1 for entry and 0.2 for removal) was applied in the analysis to identify the most explanatory subset for symptom improvement (Xu et al., 2021). To ensure that no multicollinearity existed between the explanatory variables, a collinearity test was conducted in the model.

## 3 RESULTS

### 3.1 Demographic and clinical results

There were no differences in demographic characteristics between HCs and IA adolescents at baseline (*p* > .05; Table 1). The IAT score of subjects with IA was significantly higher than that of HCs at baseline (*p* < .001) and then markedly decreased after 40 days of acupuncture treatment (*p* < .001).

**TABLE 1:** Demographical and clinical results (mean ± SD).

		Subjects with IA (*n* = 27)	
Terms	HCs (*n* = 30)	Pre‐treatment	Post‐treatment
Age (years)	21.73 ± 2.08	22.44 ± 2.42^a^	22.44 ± 2.42
Gender (male/female)	22/8	20/7^a^	20/7
Internet Addiction Test score	29.90 ± 7.18	65.26 ± 14.36^b^	45.85 ± 12.37^c^

Abbreviations: IA, internet addiction; HCs, healthy controls.

^a^ Comparison between the IA and HC groups at baseline, *p* > .05.

^b^ Comparison between the IA and HC groups at baseline, *p* < .001.

^c^ Comparison between post‐ and pretreatment in the IA group, *p* < .001.

### 3.2 FC network analysis

#### 3.2.1 Individuals with IA versus HCs at baseline

**FIGURE 2:**
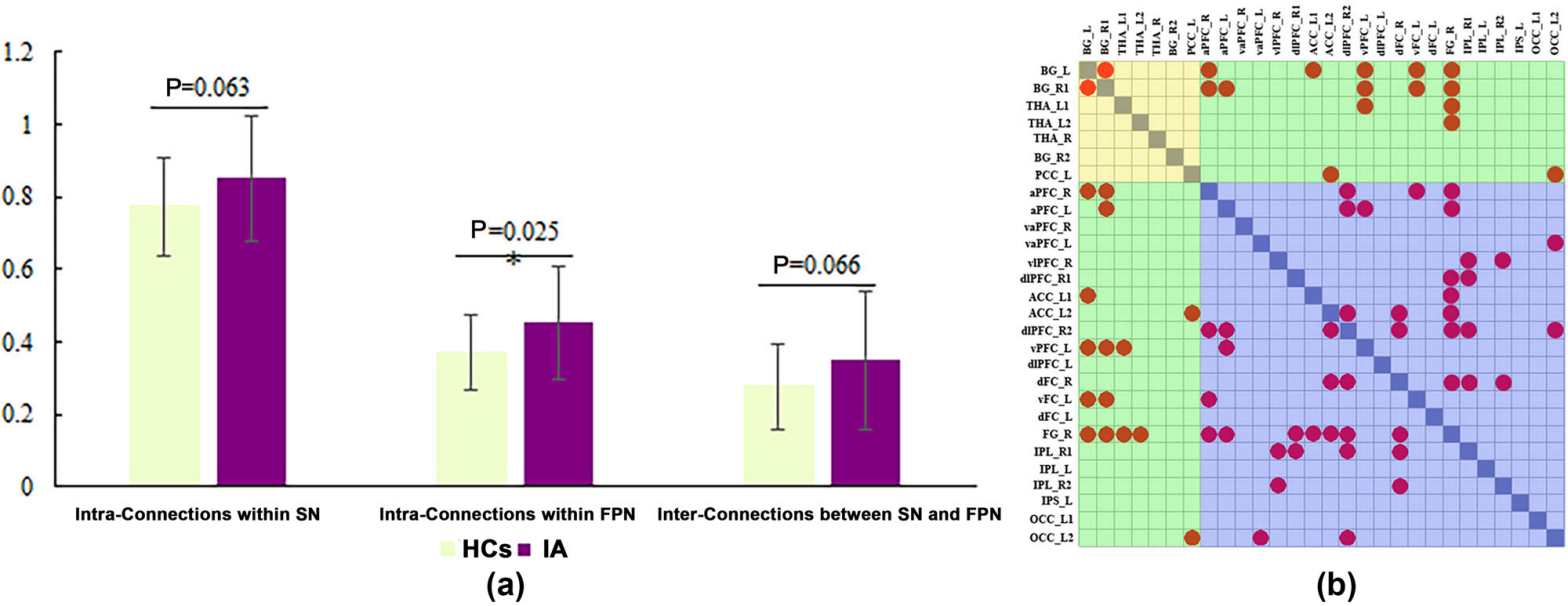
The increased functional connectivity (FC) and average FC strength among the subcortical nucleus (SN) and fronto‐parietal network (FPN) in individuals with internet addiction (IA) at baseline. (a) The average FC strength within and between the SN and FPN in healthy controls (HCs) and the IA group; (b) the significantly increased FC among the SN and FPN in subjects with IA . The yellow shadow in (b) represents the FC within the SN, the blue shadow represents the FC within the FPN, and the green shadow represents the FC between the SN and FPN. The red nodes in (b) represent the significantly heightened FC in the IA group.

**FIGURE 3:**
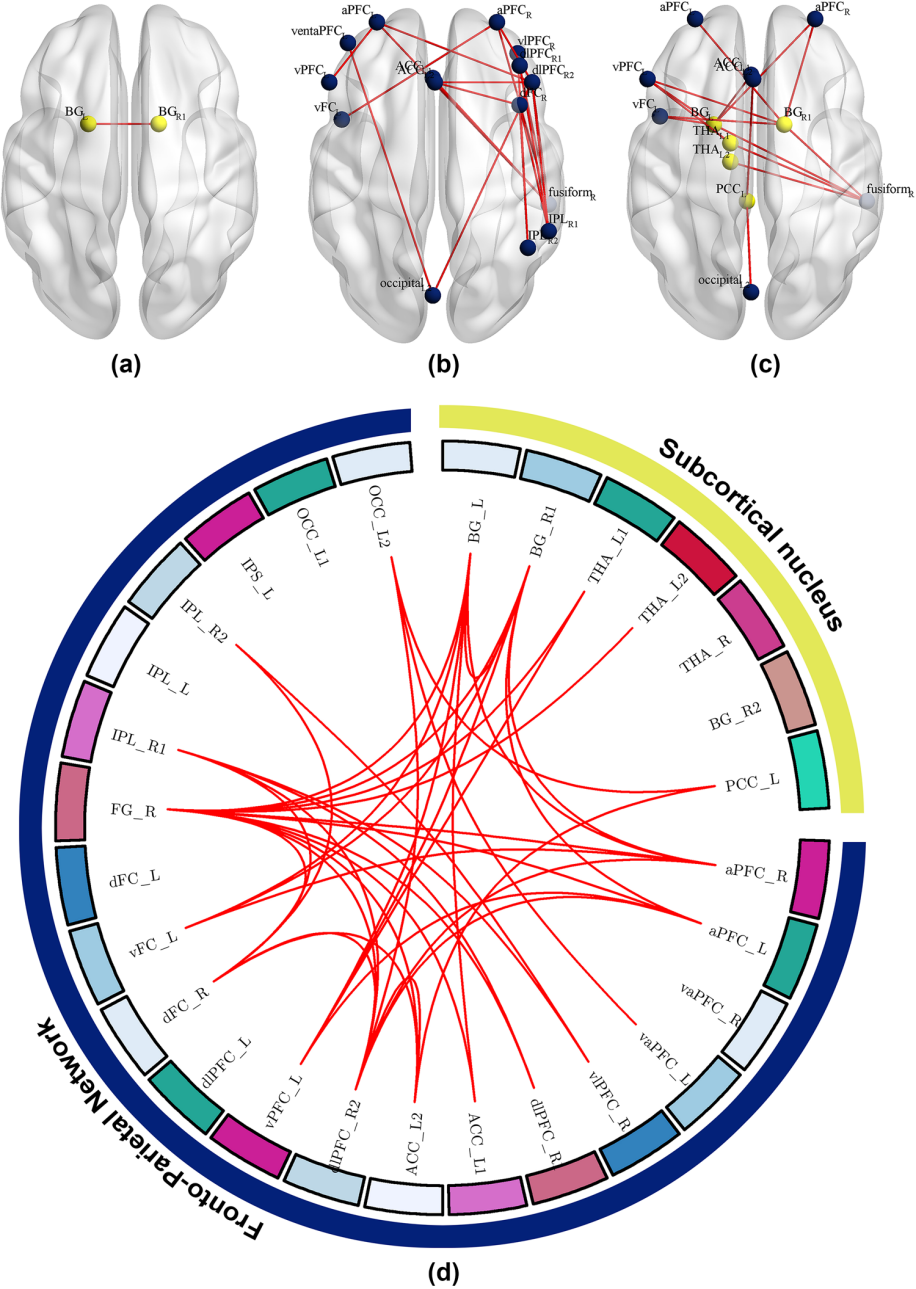
The increased functional connectivity (FC) network in internet addiction (IA) individuals at baseline. Compared with healthy controls (HCs), subjects with IA showed the (a) heightened FC within the subcortical nucleus (SN), (b) greater FC within the fronto‐parietal network (FPN), and (c) enhanced FC between the SN and FPN. (d) The summary of the abnormal FC network between HCs and individuals with IA. The yellow nodes in (a–c) and the yellow belt in (d) represent the SN regions. The blue nodes in (a–c) and the blue belt in (d) represent the FPN regions. The red lines between the nodes in (a–d) represent the increased FC in the IA group. ACC, anterior cingulate cortex; aPFC, anterior prefrontal cortex; BG, basal ganglia; dFC, dorsal frontal cortex; dlPFC, dorsolateral prefrontal cortex; FG, fusiform gyrus; IPL, inferior parietal lobule; OCC, occipital cortex; PCC, posterior cingulate cortex; THA, thalamus; vFC, ventral frontal cortex; vlPFC, ventrolateral prefrontal cortex; vPFC, ventral prefrontal cortex.

At baseline, the average FC strength within the FPN was significantly higher in individuals with IA than in HCs (*p* < .05; Figure 2a). The subjects with IA also exhibited slightly heightened average FC strength within the SN and between the SN and FPN (*p* > .05; Figure 2a). Figure 2b and Figure 3 show that the specific FC enhanced in individuals with IA(*p* < .05, NBS corrected). Compared with HCs, one of the 21 FC within the SN, 22 of the 210 FC within the FPN, and 15 of the 147 FC between the SN and FPN were significantly heightened in the IA group.

#### 3.2.2 The modulatory effects of acupuncture on IA

**FIGURE 4:**
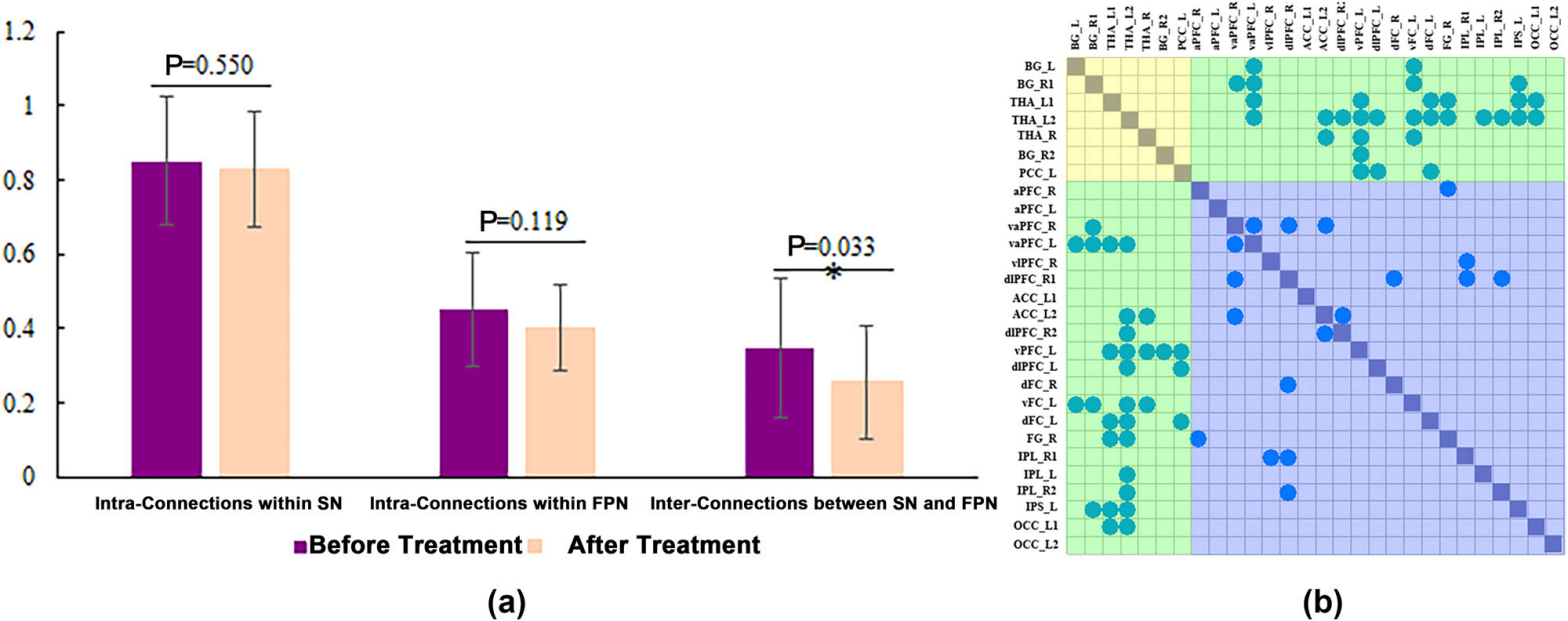
The modulations of acupuncture on the functional connectivity (FC) and average FC strength among the subcortical nucleus (SN) and fronto‐parietal network (FPN) in internet addiction (IA) subjects. (a) The average FC strength within and between the SN and FPN before and after acupuncture in IA group; (b) the significantly decreased FC among the SN and FPN in the IA group after acupuncture. The yellow shadow in (b) represents the FC within the SN, the blue shadow represents the FC within the FPN, and the green shadow represents the FC between the SN and FPN. The azure nodes in (b) represent the significantly decreased FC after acupuncture.

**FIGURE 5:**
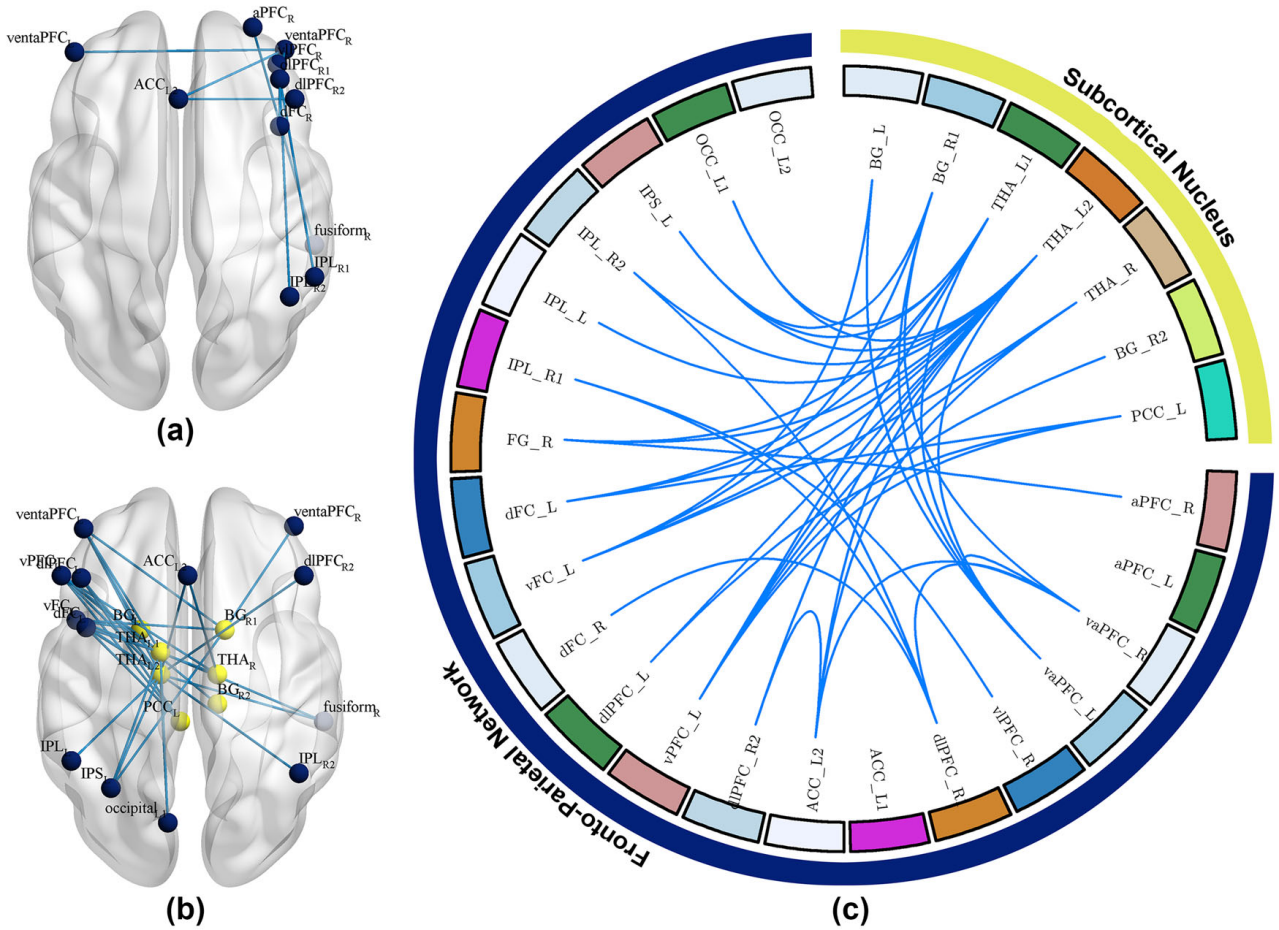
The decreased functional connectivity (FC) network in subjects with internet addiction (IA) after acupuncture. Compared with pre‐treatment, the subjects with IA after acupuncture exhibited the (a) decreased FC within the fronto‐parietal network (FPN) and (b) decreased FC between the subcortical nucleus (SN) and FPN. (c) The summary of the decreased FC network by acupuncture. The yellow nodes in (a and b) and the yellow belt in (c) represent the SN regions. The blue nodes in (a and b) and the blue belt in (c) represent the FPN regions. The azure lines between the nodes in (a–c) represent the decreased FC after treatment. ACC, anterior cingulate cortex; aPFC, anterior prefrontal cortex; BG, basal ganglia; dFC, dorsal frontal cortex; dlPFC, dorsolateral prefrontal cortex; FG, fusiform gyrus; IPL, inferior parietal lobule; OCC, occipital cortex; PCC, posterior cingulate cortex; THA, thalamus; vFC, ventral frontal cortex; vlPFC, ventrolateral prefrontal cortex; vPFC, ventral prefrontal cortex.

After 40 days of acupuncture treatment, the average FC strength between the SN and FPN in the IA group was significantly decreased (*p* < .05; Figure 4a). Individuals with IA also exhibited slightly reduced average FC strength within the FPN after treatment (*p* > .05; Figure 4a). Figure 4b and Figure 5 show the specific FC reductions in individuals with IA after acupuncture (*p* < .05, NBS corrected). Compared with the timepoint before treatment, nine of the 210 FC within the FPN and 31 of the 147 FC between the SN and FPN were significantly decreased after acupuncture. No FC within the SN was found to be decreased after acupuncture.

### 3.3 Multiple linear regression

**FIGURE 6:**
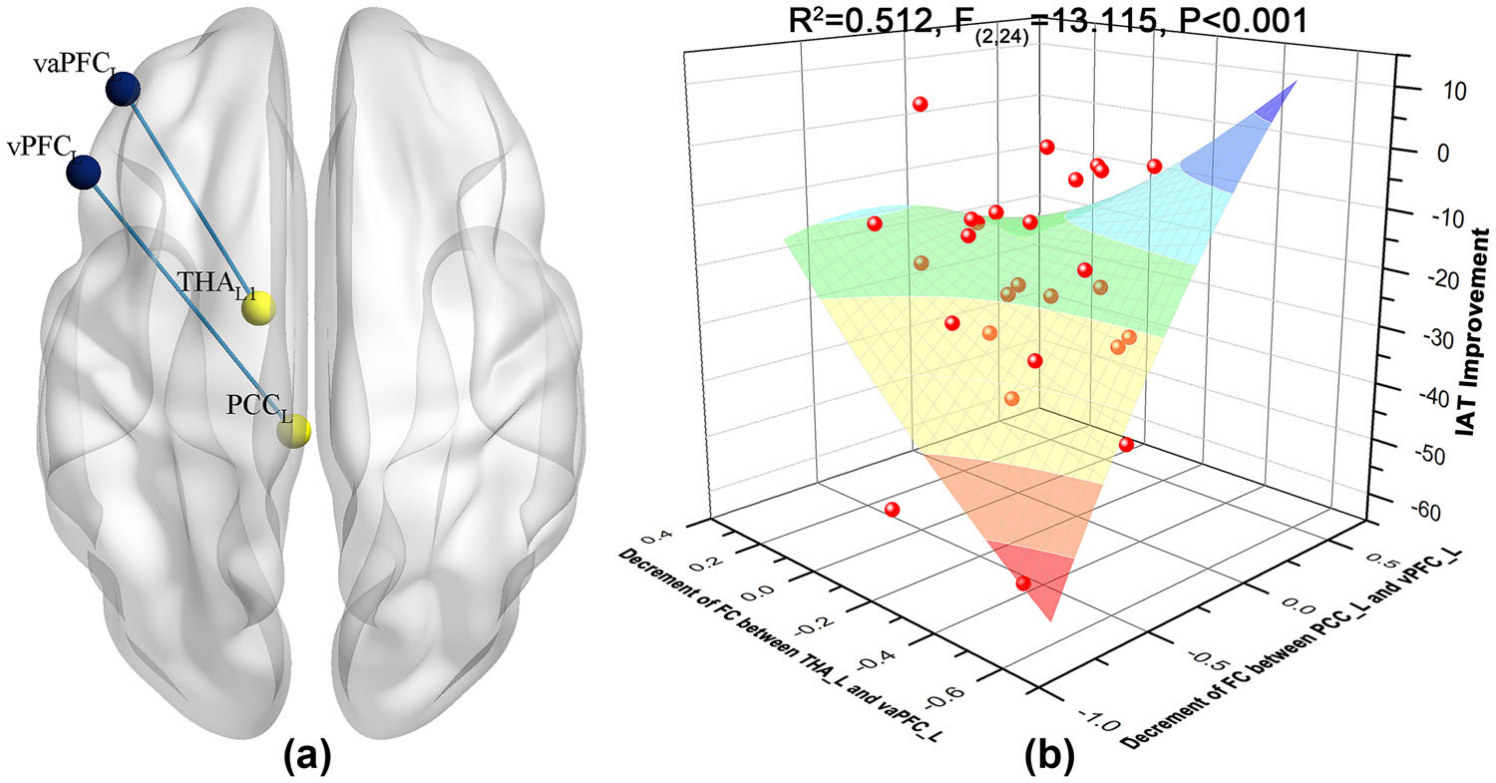
Results of the multiple linear regression model. (a) The decreases in the functional connectivity (FC) between the left midbrain and ventral prefrontal cortex and between the left thalamus and ventral anterior prefrontal cortex well fitted the IAT improvement; (b) the fitted regression surface to the Internet Addiction Test (IAT) alleviation. L, left; R, right; PCC, posterior cingulate cortex; vaPFC, ventral anterior prefrontal cortex; vPFC, ventral prefrontal cortex; THA, thalamus.

To identify the FC changes associated with symptom improvement, we further performed multiple linear regression with forward stepwise selection, which effectively predicted the improvement in IAT scores in response to acupuncture (*R* ^2^ = 0.512, *F*_(2,24)_ = 13.115, *p* < .001). As shown in Table 2 and Figure 6, IA alleviation was well fitted by the decreases in FC between the left posterior cingulate cortex (PCC) and ventral prefrontal cortex (vPFC, standardized *β* = 0.491, *p* = .04) and between the left thalamus (THA) and ventral anterior prefrontal cortex (vaPFC, standardized *β* = 0.352, *p* = .032). The PCC (MNI: −4, −31, −4) here is also termed as midbrain in Harvard‐Oxford Atlas (Desikan et al., 2006). No collinearity was observed between the selected variables.

**TABLE 2:** Functional connectivity (FC) changes associated with IAT improvement.

FC	Adjusted *β*	*t* Value	*p* Value
midbrain_L—vPFC_L	0.491	3.176	.040
THA_L—vaPFC_L	0.352	2.278	.032

*Note*: No multicollinearity problem was detected.

## 4 DISCUSSION

In the present study, we investigated the modulatory effects of acupuncture on the FC among the SN and FPN in IA. The present study revealed abnormal functional interactions among the SN and FPN in individuals with IA and the regulatory effects of acupuncture on these interactions. In comparison to HCs, individuals with IA showed significantly heightened FC within and between the SN and FPN, emphasizing the common neuropathogenesis shared by IA and substance addiction. After 40 days of acupuncture treatment, the functional interactions within the FPN and between the SN and FPN were significantly decreased in individuals with IA, along with a decrease in IAT scores. By means of multiple linear regression with forward stepwise selection, we further explored the association between IA relief and functional changes caused by acupuncture. The IAT improvement was well fitted by the decrease in FC between the left THA and vaPFC and between the left midbrain and vPFC, linking IA relief to the modulatory effect of acupuncture on the aberrant FC among the SN and FPN.

In the dual systems model, dysregulated interactions between the reward process and cognitive control have been proposed as the main characteristics of addiction (Hobkirk et al., 2019; Shulman et al., 2016; Wei et al., 2017; Zilverstand et al., 2018). The SN, which evolved from the ancient paleoencephalon (Northcutt, 2001), are considered responsible for primitive instincts, such as reward and craving, in human beings. The FPN, containing the frontal and parietal lobes, is believed to be responsible for various advanced functions, including decision‐making and cognitive control (Cole et al., 2013; Jung et al., 2019; X. Yang et al., 2019). The critical role of abnormal FC among the SN and FPN in substance addiction has been extensively investigated. Compared with nonsmokers, nicotine addicts exhibited increased coupling within the FPN, which is considered a biomarker for nicotine dependence (Janes et al., 2012). Similarly, the SN was also demonstrated to be excessive coupling in substance addiction, reflecting the dysregulation of the dopaminergic pathway in the brain (Ravichandran et al., 2021). In addition, altered FC between the SN and FPN was found to correlate with addictive symptoms, highlighting the importance of SN‐FPN FC abnormalities in addictions (Contreras‐Rodriguez et al., 2017). Given the vital role of abnormal FC among the SN and FPN in substance addiction, mounting research has further focused on the modulatory effects of related interventions on these aberrant functional interactions. A multitude of studies suggested that the altered FC within and between the SN and FPN could be normalized by drug and nondrug interventions (Garland & Howard, 2018; Konova et al., 2013). Overall, excessive interactions among the SN and FPN play a pivotal role in substance addiction, and the decoupling of these structures might be the critical target for addiction treatments.

Similar results have been reproduced in IA studies. Excessive interactions among the SN and FPN have been demonstrated in IA, indicating the shared neural abnormalities underlying IA and substance addictions (J.‐T. Zhang et al., 2018). In addition, recent research found that excessive coupling strength of the SN and FPN in internet addicts could be regulated by related treatments, implying that this coupling is a potential therapeutic target for IA (Y. Wang et al., 2020; J.‐T. Zhang et al., 2018). In the current study, we also demonstrated an increase in connections within and between the SN and FPN in IA, strengthening the evidence that IA and substance addiction share a common neural mechanism. Furthermore, the current study observed decoupling within the FPN and between the SN and FPN after acupuncture, implying that the modulatory effects of acupuncture are similar to those of other addiction therapies.

Furthermore, IA alleviation was explained well by the significant FC decreases between the SN and FPN. We adopted multiple linear regression to develop a fitting model, setting the FC decreases as explanatory variables and the IAT improvement as the target variable. In multiple linear regression modeling, forward stepwise selection was implemented to screen the potential variables to obtain a final model. As a greedy algorithm, forward stepwise selection added the fMRI signals in order of explanatory power and ultimately produced the most explanatory subset for symptom improvement (Vollering et al., 2019). In the best‐fitting model, the decrease in FC between the left THA and vaPFC and between the left midbrain and vPFC accurately predicted the IAT improvement. The midbrain is the core of the mesolimbic dopamine system and is responsible for dopamine secretion and release in the brain. The thalamus, receiving dopaminergic projections from the midbrain and projecting to the prefrontal cortex (PFC), is a key transit point in the mesolimbic dopamine pathway. Both the vPFC and vaPFC, another two regions highlighted by our model, are critical nodes in the mesocortical dopamine pathway (Poletti & Bonuccelli, 2012). In most existing theories of addiction, both dopamine pathways are greatly emphasized in addictive processes, being fundamental not only to reward motivation but also to condition enforcement (Wise, 2008). In IA, numerous studies have revealed dysfunction of the mesolimbic and mesocortical dopamine pathways (Kim & Kang, 2018; W. Zhou et al., 2021). Using the FC decreases between the left THA and vaPFC and between the left midbrain and vPFC as explanatory variables, our multiple regression model provided a good fit for the symptom improvement caused by acupuncture. These findings, consistent with the existing dopaminergic theory of addiction (Pascoli et al., 2018; Solinas et al., 2019), link symptom improvement to the modulatory effects of acupuncture on the FC among the SN and FPN, which might represent the potential neural mechanism underlying acupuncture alleviating IA.

## 5 CONCLUSIONS

In summary, the current research verified the modulatory effects of acupuncture on enhanced FC among the SN and FPN in individuals with IA. The FC changes caused by acupuncture well fitted the symptom improvement. These findings might partly reflect the neural mechanism of acupuncture on IA.

## 6 LIMITATIONS

There are several potential limitations in our study. First, all participants were recruited from universities, which may restrict the generalization of the results. Second, to recruit more subjects, all types of IA were included in this study. Further research on specific IA subtypes (e.g., social media addiction) is needed in the future. In addition, we used only a single imaging modality; multimodal neuroimaging images may provide more comprehensive data and can be included in future research. Lastly, evidence from a larger sample is still needed to validate our findings.

## AUTHOR CONTRIBUTIONS

**Yang Wang**: Writing—original draft. **Yun Qin**: Formal analysis. **Hui Li**: Writing—review & editing. **Dezhong Yao**: Conceptualization; Methodology. **Bo Sun**: Formal analysis. **Jinnan Gong**: Formal analysis. **Yu Dai**: Data curation. **Chao Wen**: Data curation. **Lingrui Zhang**: Data curation. **Chenchen Zhang**: Data curation. **Cheng Luo**: Conceptualization; Methodology. **Tianmin Zhu**: Conceptualization; Methodology.

## CONFLICT OF INTEREST STATEMENT

The authors declare no conflict of interest.

### PEER REVIEW

The peer review history for this article is available at https://publons.com/publon/10.1002/brb3.3241.

## Supporting information



Supplementary Figure

Supplementary Table 1 The locations of acupoints and the depths of needle insertion.

## Data Availability

The data sets analyzed in this research can be obtained from the corresponding author upon reasonable request for scientific research purpose.
