# Supplementary figures and images for: Acupuncture modulates the functional connectivity among the subcortical nucleus and fronto‐parietal network in adolescents with internet addiction
Source: Brain Behav. 2023 Sep 18;13(11):e3241. doi: 10.1002/brb3.3241 (PMC10636388; doi:10.1002/brb3.3241)

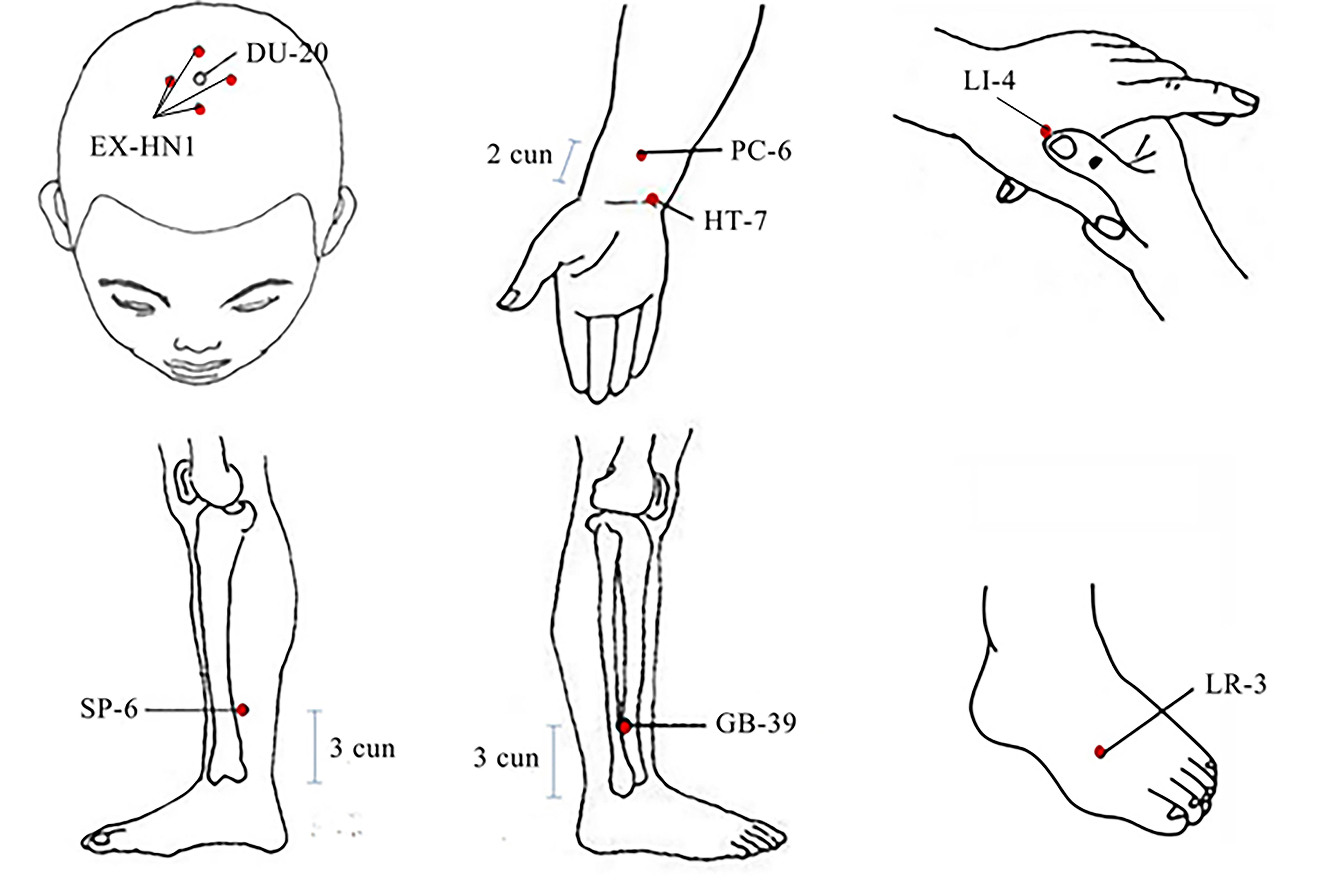

Supplement: Supplementary file 1 — Supplementary Figure [file BRB3-13-e3241-s002.jpg]
