# Supplementary material for: Acupuncture modulates the functional connectivity among the subcortical nucleus and fronto‐parietal network in adolescents with internet addiction
Source: Brain Behav. 2023 Sep 18;13(11):e3241. doi: 10.1002/brb3.3241 (PMC10636388; doi:10.1002/brb3.3241)
Supplement: Supplementary file 2 — Supplementary Table 1 The locations of acupoints and the depths of needle insertion. [file BRB3-13-e3241-s001.docx]

| Acupoints | Locations | Depth of insertion |
| --- | --- | --- |
| Baihui  (DU-20) | On the head, 5 cun directly above the midpoint of the anterior hairline. | 0.5 to 0.8 cun |
| Sishenchong (EX-HN1) | At the vertex of the head, four points in total, 1 cun repectively anterior, posterior and lateral to Baihui. | 0.5 to 0.8 cun |
| Sanyinjiao  (SP-6) | On the inner side of the calf, 3 cun above the tip of the medial malleolus Posterior border of the medial border of the tibia. | 1.0 to 1.5 cun |
| Neiguan  (PC-6) | Between the tendons of palmaris longus and flexor carpi radialis, 2 cun above the transverse crease of the wrist. | 0.5 to 1.0 cun |
| Taichong  (LR-3) | On the dorsum of the foot, in the depression proximal to the first metatarsal space. | 0.5 to 1.0 cun |
| Shenmen  (HT-7) | On the palmar ulnar end of the transverse crease of the wrist, and on the radial side of the tendon of the Flexor carpi ulnaris. | 0.3 to 0.5 cun |
| Xuanzhong (GB-39) | On the lateral side of the lower leg, 3 cun above the tip of the lateral malleolus, on the anterior border of the fibula. | 0.5 to 0.8 cun |
| Hegu  (LI-4) | Between the first and second metacarpal bones, and in the midpoint of the radial side of the second metacarpal bone. | 0.5 to 1.0 cun |

Supplementary Table 1 The locations of acupoints and the depths of needle insertion.
